# Rural-to-urban migration and risk of hypertension: longitudinal results of the PERU MIGRANT study

**DOI:** 10.1038/jhh.2015.124

**Published:** 2016-02-11

**Authors:** A Bernabe-Ortiz, J F Sanchez, R M Carrillo-Larco, R H Gilman, J A Poterico, R Quispe, L Smeeth, J J Miranda

**Affiliations:** 1 CRONICAS Center of Excellence in Chronic Diseases, Universidad Peruana Cayetano Heredia, Lima, Peru; 2 Faculty of Epidemiology and Population Health, London School of Hygiene and Tropical Medicine, London, UK; 3 US Naval Medical Research Unit No. 6 (NAMRU-6), Lima, Peru; 4 Department of International Health, Bloomberg School of Public Health, Johns Hopkins University, Baltimore, MD, USA; 5 Department of Medicine, School of Medicine, Universidad Peruana Cayetano Heredia, Lima, Peru

## Abstract

Urbanization can be detrimental to health in populations due to changes in dietary and physical activity patterns. The aim of this study was to determine the effect of migration on the incidence of hypertension. Participants of the PERU MIGRANT study, that is, rural, urban and rural-to-urban migrants, were re-evaluated after 5 years after baseline assessment. The outcome was incidence of hypertension; and the exposures were study group and other well-known risk factors. Incidence rates, relative risks (RRs) and population attributable fractions (PAFs) were calculated. At baseline, 201 (20.4%), 589 (59.5%) and 199 (20.1%) participants were rural, rural-to-urban migrant and urban subjects, respectively. Overall mean age was 47.9 (s.d.±12.0) years, and 522 (52.9%) were female. Hypertension prevalence at baseline was 16.0% (95% confidence interval (CI) 13.7–18.3), being more common in urban group; whereas pre-hypertension was more prevalent in rural participants (*P*<0.001). Follow-up rate at 5 years was 94%, 895 participants were re-assessed and 33 (3.3%) deaths were recorded. Overall incidence of hypertension was 1.73 (95%CI 1.36–2.20) per 100 person-years. In multivariable model and compared with the urban group, rural group had a greater risk of developing hypertension (RR 3.58; 95%CI 1.42–9.06). PAFs showed high waist circumference as the leading risk factor for the hypertension development in rural (19.1%), migrant (27.9%) and urban (45.8%) participants. Subjects from rural areas are at higher risk of developing hypertension relative to rural–urban migrant or urban groups. Central obesity was the leading risk factor for hypertension incidence in the three population groups.

## Introduction

Hypertension is a major problem worldwide, including low- and middle-income countries (LMIC).^1^ More than 60% of deaths due to non-communicable diseases (NCDs) are attributable to preventable cardiometabolic factors, with high blood pressure having the largest effect.^2, 3^ With globalization and unplanned urbanization, population aging, smoking, sedentary lifestyles and dietary patterns are responsible for the high prevalence of hypertension.^4^

The urbanization process is occurring at an increasing rate, especially in LMIC.^5^ Rural-to-urban migration is one of the key drivers of urbanization in LMIC. Urbanization can be detrimental to population health due to changes in diet and physical activity patterns, with a consequent increase in obesity, type 2 diabetes and cardiovascular disease.^6^ As a result, there is a need to better understand the impact of urbanization on NCDs.

A previous systematic review found that most of the reports assessing the impact of migration on cardiovascular health were cross-sectional in nature,^7^ with scarce information from longitudinal studies.^8, 9, 10, 11, 12^ Moreover, these latter papers focused on changes in blood pressure instead of the occurrence of hypertension over time. Given migration and urbanization in LMIC are linked to NCDs, an additional limitation of the available prospective designs is the small number of migrants evaluated. Peru offers a unique opportunity to assess the potential impact of within-country rural-to-urban migration on cardiovascular health since migration patterns have changed due to political violence during 1970–1990,^13^ resulting in several deaths and large numbers of displaced people.^14^ Therefore, migration was largely driven by the need to escape from armed conflicts rather than for economic reasons.

Given the aforementioned framework, we hypothesized that the rural-to-urban migrant group will have higher mean blood pressure, and for instance, higher rates of hypertension than their rural peers, yet not as high as their urban counterparts. Therefore, the aims of this study were two-fold. First, we sought to determine the effect of rural-to-urban migration on the incidence of hypertension. Second, we aimed to compare the role of potential risk factors on the occurrence of hypertension among study population groups.

## Materials and methods

The data came from the first follow-up assessment of the PERU MIGRANT study,^15^ an ongoing prospective cohort designed to assess the magnitude of differences among rural, rural-to-urban migrant and urban groups in specific cardiovascular risk factors.

Two different settings were considered for this study. San Jose de Secce, a village located in Ayacucho, was selected as the rural study site. Ayacucho, an Andean region, was one of the most affected areas during the period of conflict (1988–1993) in Peru.^16^ The area ‘Las Pampas de San Juan de Miraflores' in Lima, the capital of Peru, was selected as the urban area for the study. Both urban and rural-to-urban migrant participants were selected from this periurban area in southern Lima.

At baseline, the study groups were defined using a single random sampling of participants aged 30 years and over from the rural site of Ayacucho, the urban site of Lima, and rural-to-urban migrants from Ayacucho now residing in Lima. Information regarding selection criteria, sample size and participation rates have been published elsewhere.^17^ For this evaluation, the participants were re-contacted in the same setting where they were originally enrolled. We did not collect information about moving back to rural areas. Since participants were re-contacted where they were at baseline, we assumed that they did not move and particularly the migrant group did not go back to their rural birthplace.

The exposure of interest was migration, defined as groups of rural, migrant and urban participants according to their baseline values. The following exposures were also assessed at baseline as part of this analysis: binge drinking, defined as two or more nights of alcohol consumption in the month before the assessment and having ever drunk six or more drinks at a time; current daily smoking, defined as a self-report of smoking ⩾1 cigarettes per day; low physical activity levels defined in accordance with the International Physical Activity Questionnaire (IPAQ) protocol; thus, the categorical physical levels were coded based on total number of days of physical activity and metabolic equivalent (MET) in minutes/week;^18^ high total cholesterol, defined as fasting total cholesterol ⩾200 mg dl^−1^;^19^ obesity, defined as body mass index (BMI) ⩾30 kg m^−^^2^; high waist circumference, defined according to International Diabetes Federation cutoffs for South American populations;^20^ and type 2 diabetes, defined as any of the following conditions: fasting glucose ⩾126 mg dl^−1^, self-reported physician diagnosis and currently receiving anti-hyperglycemic drugs.^21^ Baseline fasting blood samples were obtained and analysed in a single facility, and the quality of assays was checked using regular external standards and internal duplicate assays monitored by Bio-Rad (www.biorad.com). Total cholesterol was measured in the serum, whereas glucose was measured in plasma using an enzymatic colorimetric method (GOD-PAP; Modular P-E/Roche-Cobas, Grenzach-Whylen, Germany).

Other variables of interest, also assessed at baseline were included as potential confounders as follows: sex, age (30–49 and 50+ years), education level (none/some primary school, complete primary and some secondary) and socioeconomic status according to a wealth index based on assets and household facilities and categorized separately into tertiles for study group and merged into a single variable.^22^

The outcome of interest was the occurrence of hypertension, defined as the presence of high blood pressure (systolic blood pressure ⩾140 mm Hg or diastolic blood pressure ⩾90 mm Hg) according to international guidelines^23^ or current use of anti-hypertensive medication prescribed by a physician. Blood pressure was assessed in triplicate, after a 5-min resting period, using an automatic monitor OMROM HEM-780 (OMRON, Tokyo, Japan) previously validated for adult population.^24^ In addition, pre-hypertension was defined as a systolic blood pressure from 120 to 139 mm Hg or diastolic blood pressure from 80 to 89 mm Hg.

Participants originally enrolled in the PERU MIGRANT study from 2007 to 2008 were re-contacted from 2012 to 2013 in the same setting where they were enrolled at baseline. After oral consent, the participants were asked to respond to a detailed questionnaire. The fieldworkers in rural areas were fluent in Spanish and Quechua, and they administered the survey to those with poor literacy. Weight and waist circumference were measured in triplicate by fieldworkers using standardized techniques.

Statistical analysis was conducted in STATA 13 for Windows (STATA Corp, College Station, TX, USA). The population characteristics were tabulated according to study group at baseline. Chi-squared test was used to compare categorical variables, whereas continuous variables were compared using analysis of variance test. Incidence rates per 100 person-years of follow-up and 95% confidence intervals (95%CI) were calculated, excluding those having hypertension at baseline. Incidence estimates were obtained by potential risk factors and study groups. Generalized linear models, assuming a Poisson distribution, were utilized to determine the strength of association, that is, relative risks (RRs), between the study group exposures and hypertension, controlling for several potential confounders. In addition, *post hoc* analyses were also performed in the migrant group using only migration surrogates (age at migration and years lived in urban area). Crude and adjusted models were also generated to determine RR of well-established risk factors for hypertension by study group. Given the number of confounder variables, variance inflation factor was used to determine collinearity. Finally, the population attributable fractions (PAF) were determined by using the *punaf* command for STATA.^25^

Ethical approval for the baseline and follow-up phase was granted by the Institutional Review Board at Universidad Peruana Cayetano Heredia in Lima, Peru. Participants provided verbal informed consent because of major illiteracy rates, especially in rural areas.

## Results

At baseline, data from 988 participants were analysed, mean age was 47.9 (s.d.: 12.0) years, 522 (52.8%) were females. In the study groups, 201 (20.4%), 589 (59.5%) and 199 (20.1%) were rural, rural-to-urban migrant and urban participants, respectively. Regarding the migrant group, mean age at first migration was 14.7 (s.d.: 9.0) years; in addition, mean time lived in an urban area was 32.0 (s.d.: 10.5) years. The characteristics of the study population according to study group are shown in Table 1. The overall prevalence of hypertension was 16.1% (159/988; 95%CI 13.8–18.4), and prevalence estimates varied by study group: hypertension was more common among the urban population, but pre-hypertension was more prevalent among the rural group (*P*<0.001). Moreover, of all participants with hypertension, 50% of rural dwellers, 77.6% of migrants and 47.5% of urban individuals were previously diagnosed by a physician (*P*=0.001).

Of the 988 participants enrolled at baseline, 60 (6.1%) were lost to follow-up, and 33 (3.3%) died. Thus, of the 895 (90.6%) re-contacted, 133 (14.9%) were further excluded from incidence calculations due a hypertension diagnosis at baseline. The mean time of follow-up was 5.2 (s.d.: 0.6) years, completing a total of 3962 person-years of follow-up. A total of 66 new cases of hypertension were identified, leading to an overall incidence of 1.73 (95%CI 1.36–2.20) per 100 person-years (5-year cumulative incidence: 8.65%). In the follow-up the systolic and diastolic blood pressure means were similar across study groups (Supplementary E-Table 1). The incidence in the rural group was 2.44 (95%CI 1.62-3.67), the incidence in the rural-to-urban migrant group was 1.60 (95%CI 1.15-2.22), and the incidence in the urban group was 1.11 (95%CI 0.53-2.33) (*P*<0.001) In addition, of all new cases, 91.3% of rural dwellers, 75.0% of migrants and 100% of urban individuals reported to be diagnosed of hypertension by a physician in the previous 5 years of the follow-up (*P*=0.19). The incidences of hypertension according to population characteristics and study group at baseline is shown in Table 2. Notably the incidence of hypertension due to pre-hypertension was highest among rural than migrant or urban participants.

Compared with the urban group, the rural participants had a higher risk of hypertension, and the magnitude of RR increased with further adjustment. After controlling for demographic and behavioural confounders and compared with the urban group, rural participants were four times more likely to develop hypertension (RR 3.58; 95%CI 1.42–9.06). The migrant group was also at a high risk of hypertension; however, results were not significant. Details are shown in Table 3. Using data from migration surrogates and after controlling for several confounders, those migrants living 30 years or more in the urban setting were at lower risk of hypertension when compared with those living less than 30 years; however, results were not significant (RR 0.85; 95%CI 0.35–2.03). Similarly, when the age at migration was used, those who reported migrating at 15 years or over were at greater risk of developing hypertension when compared with those migrating at age below 15 years, but results were not significant (RR 1.04; 95%CI 0.53–2.04).

As shown in Table 4, in the multivariable models, current daily smoking (RR 4.26) and high waist circumference (RR 2.68) were found to be associated with a higher risk of hypertension among the rural group; and only pre-hypertension increased the risk among the migrant population (RR 2.98). Similarly, only type 2 diabetes at baseline (RR 7.10) increased the risk of hypertension among urban population.

PAFs were also calculated for each study group (Supplementary E-Table 2) and showed considerable heterogeneity across study groups. Many of the estimates were under 10% in the rural and migrant groups, except for high waist circumference (19.1 and 27.9%, respectively) and pre-hypertension (31.3 and 40.6%, respectively). In contrast, obesity, high waist circumference, type 2 diabetes and pre-hypertension were markedly high in the urban group (52.6, 45.8, 24.5 and 37.9%, respectively).

## Discussion

This prospective ongoing cohort study included different study groups and was explicitly designed to ascertain whether rural-urban migrant and non-migrant groups have differential risks of NCDs. The risk of hypertension was almost four times greater among rural residents relative to their urban counterparts. Although the migrant group had also an increased risk compared with urban individuals, this finding was not significant. Factors associated with the incidence of hypertension in the multivariate model differed by study group. High waist circumference and daily smoking were highest in the rural group, pre-hypertension was highest in the rural-to-urban migrant group, and type 2 diabetes was highest in the urban group. In addition, using estimates of PAFs, obesity-related markers (i.e., BMI, but especially high waist circumference) were the leading factors that increased the risk of hypertension in the three population groups, particularly among urban individuals.

Our results consistently indicated a higher risk of hypertension among the rural groups compared with the urban groups. This observation is supported by the high proportion of rural pre-hypertensive individuals at baseline but also the high incidence rate of hypertension during follow-up period. Although hypertension was markedly higher in the urban group, at baseline, one-third of the rural and migrant populations were pre-hypertensive. This information highlights the diversity of scenarios for hypertension, revealing major risks in specific groups. This approach of identifying different risk magnitudes in low-resource settings suggests that other non-communicable conditions may also have similar complexities, particularly in many LMIC in (epidemiological and nutritional) transition.

High pre-hypertension rates at baseline in our rural group as well as low health standards associated with living in rural settings, such as poverty, malnutrition, poor hygiene and inadequate health care,^26^ might potentially explain these findings. Thus, these broader contextual variables applicable to rural settings, paired with ongoing nutritional transition, may potentially increase the risk of negative lifestyle consequences, particularly obesity, followed by hypertension.^27^ In addition, despite controlling for different different sociodemographic and lifestyles factors, our study lacked information about dietary patterns, a key determinant of cardiovascular outcomes.^28^ Diets in Andean region populations, such as those in our study, have changed over time. The consumption of sugar and cholesterol intake have increased, whereas vegetable, starchy root and fruit intake has considerably reduced.^29^ Because people living in rural areas have much higher levels of physical activity as shown in this study and a previous report,^30^ we believe that much of the effect observed in this study might be explained by diet. However, further studies are needed to evaluate the impact of diet on hypertension in these study groups.

Since the urbanization phenomenon in rural areas dates back to the last decades, another possible explanation for the higher hypertension incidence in the rural group is a period effect: higher incidence of hypertension in this population is a rather new feature. This is further supported as rural subjects had higher prevalence of pre-hypertension. Consequently, current urbanization process is reaching out to those pre-hypertensive subjects. This gives a window opportunity to implement prevention strategies before all pre-hypertensive rural subjects meet criteria for hypertension.

Early reports on this topic were performed in 1990s in Kenya and China,^9, 31^ showing an increment of blood pressure due to rural-to-urban migration. These findings are contrary to our results perhaps because these past studies were conducted when travel and communication between rural and urban areas were more difficult and urban lifestyle was less likely adopted by rural dwellers. As a result, there is still limited longitudinal information available regarding the impact of migration on hypertension because many of the published studies reported changes in blood pressure means instead of hypertension rates.^8, 10, 11^ These studies have also compared migrants versus non-migrant groups, instead of urban, rural-to-urban migrants, and rural populations. As a result, our findings expand on previous knowledge, demonstrating that the greatest risk of developing hypertension occurs among the rural population.

Migrant populations have been thought to be potentially more affected by unhealthy practices acquired from living in a new setting, especially among those who are more acculturated.^32^ However, such findings have always been controversial. For example, a previous study has suggested that some migration surrogates may directly influence broader social determinants of disease,^33^ thus reducing the possibility of acquiring cardiovascular outcomes. However, many of these findings have come from cross-sectional studies and have involved migrants who moved from developing to developed countries instead of rural-to-urban within-country migrants. In a *post hoc* analysis, we attempted to model the effect of migration surrogates (age at migration and years of urban exposure), but the results were not conclusive.

In terms of using blood pressure as a continuous variable, previous cross-sectional studies have shown non-significant differences in blood pressure levels among migrants compared with rural groups^15, 31^ and a pattern of significantly lower systolic and diastolic blood pressure in migrant compared with urban groups. Our study found differences in mean systolic and diastolic blood pressure levels by study group at baseline but not after 5 years of follow-up. Two different longitudinal studies have reported changes in blood pressure levels in migrant groups.^9, 10^ One study found that the systolic blood pressure of migrants was significantly higher than that of rural controls;^9^ whereas the other study only found elevated systolic and diastolic blood pressures in male migrants versus non-migrants.^10^

Although cardiovascular risk factors are well known, global policies require local adaptation according to population profiles. It is important to understand the local burden of disease, including the within-country heterogeneity of NCD distribution and their risk factors, to effectively prioritize adequate responses. For example, considering benefits and feasibility, reducing tobacco use is recommended as one of the best initiative.^34^ However, in our study, only a small proportion (<5%) smoked every day; thus, the potential impact of an intervention focused on this risk factor would be almost negligible as highlighted by results of PAFs.

According to our results, special attention should be focused on obesity, as BMI and waist circumference showed different distributions across the study groups. In addition, type 2 diabetes was a key factor among the urban participants and a natural consequence of the increasing burden of obesity. Our results are compatible with the need to reduce of central obesity;^10^ more than any other factor, focusing on obesity may reduce the risk of hypertension in all three populations, especially among urban people. These results also show that different regions and populations within the same country are at different stages of the nutritional transition. Therefore, interventions to prevent increasing rates of NCD in our context should be focused on reducing obesity.

Pre-hypertension deserves more attention, as it was present in almost one-third of the study participants at baseline. Interventions must be more inclusive and extensive of this stage to keep from progressing toward hypertension.^35^ According to the PAF in our results, hypertension would be reduced by 30% in all study groups if a reduction in blood pressure under 120/80 mm Hg was guaranteed.

A strength of this study includes the calculation of hypertension incidence over a 5-year period in well-defined rural, migrant and urban populations. Although the fact that our study population migrated due to different reasons than socioeconomic mobility is a novelty of the study, it could also be regarded as a limitation in terms of external generalizability. Migration is mostly driven by socioeconomic upward mobility, which comes along with different risk factors (for example, sedentarism or unhealthy diets). Our study population did not move because they had achieved better socioeconomic standards, and thus our results may not fully represent the new migration waves across the world. Other limitations should also be described. First, although the rate of attrition during follow-up was relatively low (<10%), the results might be affected by selection bias, especially in the urban setting involving data collection among both migrants and urban subjects. As previously reported,^17^ the response rates of migrants (77.7%) and urban residents (56.8%) were low in the baseline study compared with rural group (84.8%). Therefore, participants who were originally enrolled might have different characteristics than those who declined to participate. Second, although the definitions of rural, migrant and urban populations can change over time, we assumed did not occur and did not affect our estimates, because all of the participants were re-contacted in the same area where they originally were enrolled. Third, power might be an issue as many well-recognized factors were not associated with the progression towards hypertension. However, because PAFs assess the contribution of a risk factor to a disease, they can provide a better understanding of the role of these factors in the study populations. Finally, results could also reflect the effect of unmeasured confounders like chronic kidney disease. Unfortunately, we did not collect data about this condition at baseline; yet at follow-up we asked whether the participant has been diagnosed with chronic kidney disease in the past 5 years showing a prevalence of less than 1%. In addition, a previous report in the urban study area found that about 20% of the population presented some degree of chronic kidney disease, and 19% of subjects with chronic kidney disease had hypertension as well.^36^ Despite these findings, our results showed such a strong hypertension risk given the different population groups included as the exposure variable, that it is hard to think the risk would be completely explained by chronic kidney disease. The extent at which chronic kidney disease confound, or explain, the association of interest must be addressed by future studies, particularly as the burden of chronic kidney disease is rather neglected in rural resource-limited settings.

In conclusion, the incidence of hypertension was higher in rural populations than in migrant and urban groups. Risk factors for hypertension differed across study groups, and almost one-third of participants were pre-hypertensive at baseline. Obesity, assessed by waist circumference and BMI, was the leading risk factor for developing hypertension in the three groups evaluated. The results suggest that interventions to address hypertension should focus on reducing obesity, especially in urban settings.


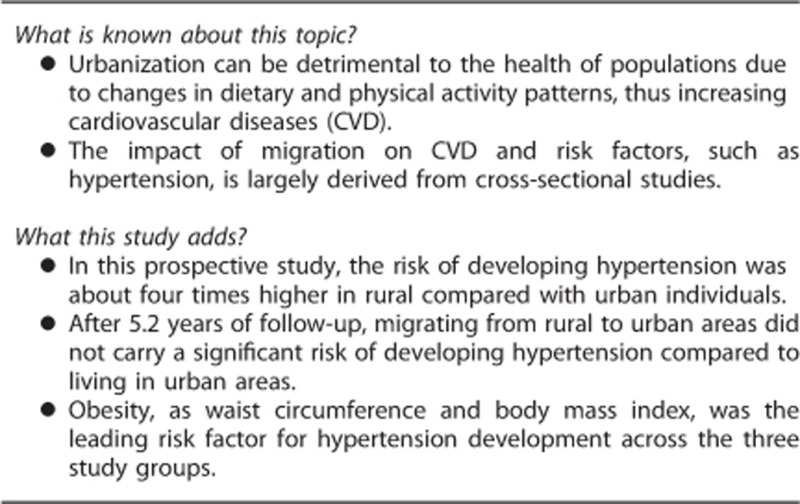


## Figures and Tables

**Table 1:** Baseline characteristics of the study sample according to study group

	*Rural group*	*Migrant group*	*Urban group*	P-*value*
	(n=*201)*	(n=*589)*	(n=*199)*	
*Sex*				
Male	95 (47.3%)	280 (47.5%)	92 (46.2%)	0.95

*Age*				
30–49 years	117 (58.2%)	337 (57.2%)	110 (55.3%)	0.57
50+ years	84 (41.8%)	252 (42.8%)	89 (44.7%)	

*Education level*				
None/some primary school	132 (65.7%)	183 (31.1%)	13 (6.6%)	<0.001
Complete primary	30 (14.9%)	99 (16.8%)	23 (11.6%)	
Some secondary	39 (19.4%)	306 (52.1%)	162 (81.8%)	

*Socioeconomic status*				
Lowest tertile	196 (97.5%)	119 (20.2%)	32 (16.1%)	<0.001
Middle tertile	5 (2.5%)	253 (43.0%)	69 (34.7%)	
Highest tertile	0 (0.0%)	217 (36.8%)	98 (49.2%)	

*Binge drinking*				
Yes	23 (11.4%)	47 (8.0%)	17 (8.5%)	0.32

*Current daily smoking*				
Yes	1 (0.5%)	15 (2.6%)	17 (8.5%)	<0.001

*Physical activity*				
Low levels	4 (2.05)	173 (29.7%)	78 (39.4%)	<0.001

*Total cholesterol*				
⩾200 mg dl^−1^	15 (7.5%)	220 (37.4%)	71 (35.7%)	<0.001

*High waist circumference*				
Yes	30 (15.2%)	354 (60.3%)	132 (66.7%)	<0.001

*Obesity*				
BMI⩾30 kg m^−2^	6 (3.0%)	124 (21.1%)	68 (34.2%)	<0.001

*Type 2 diabetes*				
Yes	3 (1.5%)	21 (3.6%)	16 (8.0%)	0.003

*Systolic blood pressure (mm Hg)*^a^				
Mean (s.d.)	120.9 (18.7)	119.9 (16.4)	128.2 (22.9)	<0.001

*Diastolic blood pressure (mm Hg)*^a^				
Mean (s.d.)	74.2 (9.2)	71.3 (9.3)	76.2 (11.5)	<0.001

*Blood pressure status*				
Normal	106 (52.7%)	318 (54.1%)	87 (43.7%)	<0.001
Pre-hypertension	71 (35.3%)	194 (33.0%)	53 (26.6%)	
Hypertension	24 (11.9%)	76 (12.9%)	59 (29.7%)	

Abbreviation: BMI, body mass index. Results may not add because of missing values.

a Analysis of variance test was used for comparisons, instead of Chi-squared test for categorical variables.

**Table 2:** Incidence rates and 95%CI of hypertension according to population characteristics at baseline

	*Rural group*	*Migrant group*	*Urban group*
*Sex*			
Female	2.65 (1.57–4.48)	2.14 (1.46–3.14)	1.72 (0.77–3.83)
Male	2.17 (1.13–4.18)	0.97 (0.52–1.80)	0.35 (0.05–2.52)

*Age*			
30–49 years	1.98 (1.12–3.49)	1.07 (0.65–1.77)	0.95 (0.36–2.54)
50+ years	3.27 (1.81–5.91)	2.49 (1.62–3.82)	1.42 (0.46–4.39)

*Education level*			
None/some primary school	2.23 (1.30–3.85)	2.48 (1.52–4.05)	—
Complete primary	2.47 (0.93–6.58)	1.36 (0.56–3.26)	2.22 (0.31–15.78)
Some secondary	3.03 (1.36–6.75)	1.22 (0.74–2.03)	1.10 (0.49–2.45)

*Socioeconomic status*			
Lowest tertile	1.95 (1.08–3.52)	1.57 (0.93–2.65)	1.82 (0.68–4.84)
Middle tertile	1.52 (0.21–10.76)	1.96 (1.11–3.45)	0.53 (0.075–3.76)
Highest tertile	3.53 (1.95–6.37)	1.35 (0.73–2.50)	0.90 (0.23–3.60)

*Binge drinking*			
No	2.42 (1.56–3.74)	1.68 (1.21–2.34)	1.22 (0.58–2.55)
Yes	2.63 (0.85–8.16)	0.60 (0.08–4.25)	—

*Daily smoking*			
No	2.35 (1.55–3.57)	1.65 (1.19–2.28)	1.18 (0.56–2.48)
Yes	16.67 (2.35–118.32)	—	—

*Physical activity*			
Moderate/high levels	2.47 (1.64–3.72)	1.67 (1.14–2.45)	1.32 (0.55–3.18)
Low levels	—	1.52 (0.82–2.82)	0.79 (0.20–3.16)

*Total cholesterol*			
<200 mg dl^−1^	2.18 (1.39–3.42)	1.31 (0.84–2.06)	1.02 (0.38–2.73)
⩾200 mg dl^−1^	5.56 (2.09–14.80)	2.13 (1.32–3.42)	1.25 (0.40–3.88)

*High waist circumference*			
No	2.05 (1.26–3.35)	0.89 (0.45–1.79)	0.41 (0.06–2.93)
Yes	4.67 (2.22–9.79)	2.08 (1.44–3.01)	1.56 (0.70–3.48)

*Obesity*			
BMI <30 kg m^−^^2^	2.32 (1.51–3.55)	1.45 (0.99–2.13)	0.45 (0.11–1.82)
BMI ⩾30 kg m^−2^	5.56 (1.39–22.21)	2.19 (1.18–4.07)	2.62 (1.09–6.29)

*Type 2 diabetes*			
No	2.47 (1.64–3.72)	1.52 (1.08–2.14)	0.85 (0.35–2.05)
Yes	—	3.95 (1.27–12.24)	4.55 (1.14–18.17)

*Pre-hypertension*			
No	1.74 (0.93–3.23)	0.99 (0.58–1.67)	0.76 (0.24–2.34)
Yes	3.55 (2.06–6.12)	2.66 (1.75–4.04)	1.71 (0.64–4.55)

Abbreviations: BMI, body mass index; CI, confidence interval. Incidence rate was not calculated as there were no hypertension cases during follow-up (—).

**Table 3:** Relative risks of the association between hypertension and study group: crude and adjusted models

*Study group*	*Crude model*	*Adjusted model*^a^	*Adjusted model*^b^
	*RR (95%CI)*	*RR (95%CI)*	*RR (95%CI)*
Urban	1 (Reference)	1 (Reference)	1 (Reference)
Migrant	1.44 (0.66–3.17)	1.43 (0.65–3.15)	1.56 (0.73–3.30)
Rural	2.20 (0.97–4.97)	2.30 (0.93–5.71)	**3.58 (1.42–9.06)**

Abbreviations: CI, confidence interval; RR, relative risk. Bold estimates are statistically significant (*P*<0.05).

a Adjusted by sex, age, education level and socioeconomic status.

b Adjusted by sex, age, education level, socioeconomic status, binge drinking, current daily smoking, physical activity, high total cholesterol, obesity, high waist circumference and type 2 diabetes.

**Table 4:** Factors and risk of hypertension according to study group: crude and adjusted models

	*Rural group*		*Migrant group*		*Urban group*	
	*Crude model*	*Adjusted model*^a^	*Crude model*	*Adjusted model*^a^	*Crude model*	*Adjusted model*^a^
	*RR (95%CI)*	*RR (95%CI)*	*RR (95%CI)*	*RR (95%CI)*	*RR (95%CI)*	*RR (95%CI)*
*Binge drinking*						
Yes	1.09 (0.36–3.33)	1.33 (0.43–4.14)	0.36 (0.05–2.52)	0.57 (0.08–4.11)	—	—

*Current daily smoking*						
Yes	**7.09 (4.81–10.5)**	**4.26 (1.44–12.5)**	—	—	—	—

*Physical activity*						
Low levels	—	—	0.91 (0.45–1.84)	0.87 (0.44–1.71)	0.60 (0.12–3.00)	0.90 (0.20–4.05)

*High total cholesterol*						
⩾200 mg dl^−1^	**2.54 (1.02–6.30)**	2.34 (0.84–6.52)	1.63 (0.87–3.04)	1.27 (0.65–2.47)	1.22 (0.28–5.27)	1.05 (0.24–4.55)

*Obesity*						
BMI ⩾30 kg m^−^^2^	2.40 (0.72–7.98)	2.39 (0.59–9.68)	1.51 (0.76–3.02)	1.15 (0.57–2.34)	5.76 (1.16–28.7)	3.79 (0.83–17.3)

*High waist circumference*						
Yes	**2.28 (1.04–4.97)**	**2.68 (1.22–5.89)**	**2.33 (1.09–5.00)**	1.56 (0.69–3.55)	3.78 (0.46–30.9)	2.15 (0.42–10.9)

*Type 2 diabetes*						
Yes	—	—	2.60 (0.91–7.46)	1.70 (0.59–4.86)	**5.34 (1.16–24.5)**	**7.10 (1.56–32.3)**

*Pre-hypertension*						
Yes	2.05 (0.96–4.38)	2.24 (1.00–5.02)	**2.69 (1.41–5.12)**	**2.98 (1.55–5.73)**	2.26 (0.52–9.76)	2.98 (0.74–12.1)

Abbreviations: BMI, body mass index; CI, confidence interval; RR, relative risk. Bolded estimates are significant, *P*<0.05.

a Adjusted by sex, age, education level, and socioeconomic status.

## References

[bib1] World Health Organization. Global Status Report on Non Communicable Diseases 2010. WHO: Geneva, Switzerland, 2011.

[bib2] Global Burden of Metabolic Risk Factors for Chronic Diseases Collaboration. Cardiovascular disease, chronic kidney disease, and diabetes mortality burden of cardiometabolic risk factors from 1980 to 2010: a comparative risk assessment. Lancet Diabetes Endocrinol 2014; 2(8): 634–647. 24842598 10.1016/S2213-8587(14)70102-0 PMC4572741

[bib3] Lim SS, Vos T, Flaxman AD, Danaei G, Shibuya K, Adair-Rohani H et al. A comparative risk assessment of burden of disease and injury attributable to 67 risk factors and risk factor clusters in 21 regions, 1990-2010: a systematic analysis for the Global Burden of Disease Study 2010. Lancet 2012; 380(9859): 2224–2260. 23245609 10.1016/S0140-6736(12)61766-8 PMC4156511

[bib4] Ordunez P. Cardiovascular health in the Americas: facts, priorities and the UN high-level meeting on non-communicable diseases. MEDICC Rev 2011; 13(4): 6–10. 10.37757/MR2011V13.N4.3 22143601

[bib5] Patel RB, Burke TF. Urbanization—an emerging humanitarian disaster. N Engl J Med 2009; 361(8): 741–743. 19692687 10.1056/NEJMp0810878

[bib6] Patil RR. Urbanization as a determinant of health: a socioepidemiological perspective. Soc Work Public Health 2014; 29(4): 335–341. 24871771 10.1080/19371918.2013.821360

[bib7] Hernandez AV, Pasupuleti V, Deshpande A, Bernabe-Ortiz A, Miranda JJ. Effect of rural-to-urban within-country migration on cardiovascular risk factors in low- and middle-income countries: a systematic review. Heart 2012; 98(3): 185–194. 21917659 10.1136/heartjnl-2011-300599 PMC3272377

[bib8] Poulter NR, Khaw K, Hopwood BE, Mugambi M, Peart WS, Sever PS. Determinants of blood pressure changes due to urbanization: a longitudinal study. J Hypertens Suppl 1985; 3(3): S375–S377. 2856743

[bib9] Poulter NR, Khaw KT, Hopwood BE, Mugambi M, Peart WS, Rose G et al. The Kenyan Luo migration study: observations on the initiation of a rise in blood pressure. BMJ 1990; 300(6730): 967–972. 2344502 10.1136/bmj.300.6730.967 PMC1662695

[bib10] Salmond CE, Prior IA, Wessen AF. Blood pressure patterns and migration: a 14-year cohort study of adult Tokelauans. Am J Epidemiol 1989; 130(1): 37–52. 2787109 10.1093/oxfordjournals.aje.a115321

[bib11] Unwin N, James P, McLarty D, Machybia H, Nkulila P, Tamin B et al. Rural to urban migration and changes in cardiovascular risk factors in Tanzania: a prospective cohort study. BMC Public Health 2010; 10: 272. 20497567 10.1186/1471-2458-10-272 PMC2892446

[bib12] Unwin N, McLarty D, Machibya H, Aspray T, Tamin B, Carlin L et al. Changes in blood pressure and lipids associated with rural to urban migration in Tanzania. J Hum Hypertens 2006; 20(9): 704–706. 16738685 10.1038/sj.jhh.1002056

[bib13] Durand J. The Peruvian diaspora: portrait of a migratory process. Lat Am Perspect 2010; 37(5): 12–28. 20824948 10.1177/0094582x10379103

[bib14] Barrientos Hernandez DH, Church AL. Terrorism in Peru. Prehosp Disaster Med 2003; 18(2): 123–126. 15074494 10.1017/s1049023x0000087x

[bib15] Miranda JJ, Gilman RH, Smeeth L. Differences in cardiovascular risk factors in rural, urban and rural-to-urban migrants in Peru. Heart 2011; 97(10): 787–796. 21478383 10.1136/hrt.2010.218537 PMC3183994

[bib16] Pedersen D, Tremblay J, Errazuriz C, Gamarra J. The sequelae of political violence: assessing trauma, suffering and dislocation in the Peruvian highlands. Social Sci Med 2008; 67(2): 205–217. 10.1016/j.socscimed.2008.03.040 18423959

[bib17] Miranda JJ, Gilman RH, Garcia HH, Smeeth L. The effect on cardiovascular risk factors of migration from rural to urban areas in Peru: PERU MIGRANT Study. BMC Cardiovasc Disord 2009; 9: 23. 19505331 10.1186/1471-2261-9-23 PMC2701408

[bib18] Department of Health and Human Services. 2008 Physical Activity Guidelines for Americans. Department of Health and Human Services Rockville, MD, USA, 2008.

[bib19] National Cholesterol Education Program (NCEP) Expert Panel on Detection, Evaluation, and Treatment of High Blood Cholesterol in Adults (Adult Treatment Panel III). Third Report of the National Cholesterol Education Program (NCEP) Expert Panel on Detection, Evaluation, and Treatment of High Blood Cholesterol in Adults (Adult Treatment Panel III) final report. Circulation 2002; 106(25): 3143–3421. 12485966

[bib20] Alberti KG, Eckel RH, Grundy SM, Zimmet PZ, Cleeman JI, Donato KA et al. Harmonizing the metabolic syndrome: a joint interim statement of the International Diabetes Federation Task Force on Epidemiology and Prevention; National Heart, Lung, and Blood Institute; American Heart Association; World Heart Federation; International Atherosclerosis Society; and International Association for the Study of Obesity. Circulation 2009; 120(16): 1640–1645. 19805654 10.1161/CIRCULATIONAHA.109.192644

[bib21] World Health Organization. Definition, Diagnosis and Classification of Diabetes Mellitus and its Complications. WHO: Geneva, Switzerland, 1999.

[bib22] Howe LD, Galobardes B, Matijasevich A, Gordon D, Johnston D, Onwujekwe O et al. Measuring socio-economic position for epidemiological studies in low- and middle-income countries: a methods of measurement in epidemiology paper. Int J Epidemiol 2012; 41(3): 871–886. 22438428 10.1093/ije/dys037 PMC3396323

[bib23] Chobanian AV, Bakris GL, Black HR, Cushman WC, Green LA, Izzo JL Jr. et al. The Seventh Report of the Joint National Committee on prevention, detection, evaluation, and treatment of high blood pressure: the JNC 7 report. JAMA 2003; 289(19): 2560–2572. 12748199 10.1001/jama.289.19.2560

[bib24] Coleman A, Steel S, Freeman P, de Greeff A, Shennan A. Validation of the Omron M7 (HEM-780-E) oscillometric blood pressure monitoring device according to the British Hypertension Society protocol. Blood Press Monit 2008; 13(1): 49–54. 18199924 10.1097/MBP.0b013e3282cb57b6

[bib25] Newson RB. Attributable and unattributable risks and fractions and other scenario comparisons. Stata J 2013; 13(4): 672–698.

[bib26] Gracey M, King M. Indigenous health part 1: determinants and disease patterns. Lancet 2009; 374(9683): 65–75. 19577695 10.1016/S0140-6736(09)60914-4

[bib27] Popkin BM, Adair LS, Ng SW. Global nutrition transition and the pandemic of obesity in developing countries. Nutr Rev 2012; 70(1): 3–21. 22221213 10.1111/j.1753-4887.2011.00456.x PMC3257829

[bib28] Bowen L, Ebrahim S, De Stavola B, Ness A, Kinra S, Bharathi AV et al. Dietary intake and rural-urban migration in India: a cross-sectional study. PLoS ONE 2011; 6(6): e14822. 21731604 10.1371/journal.pone.0014822 PMC3120774

[bib29] Bermudez OI, Tucker KL. Trends in dietary patterns of Latin American populations. Cad Saude Publica 2003; 19(Suppl 1): S87–S99. 12886439 10.1590/s0102-311x2003000700010

[bib30] Masterson Creber RM, Smeeth L, Gilman RH, Miranda JJ. Physical activity and cardiovascular risk factors among rural and urban groups and rural-to-urban migrants in Peru: a cross-sectional study. Rev Panam Salud Publica 2010; 28(1): 1–8. 20857014 10.1590/s1020-49892010000700001 PMC2957283

[bib31] He J, Klag MJ, Whelton PK, Chen JY, Mo JP, Qian MC et al. Migration, blood pressure pattern, and hypertension: the Yi Migrant Study. Am J Epidemiol 1991; 134(10): 1085–1101. 1746519 10.1093/oxfordjournals.aje.a116012

[bib32] Scribner R. Paradox as paradigm—the health outcomes of Mexican Americans. Am J Public Health 1996; 86(3): 303–305. 8604751 10.2105/ajph.86.3.303 PMC1380505

[bib33] Bernabe-Ortiz A, Gilman RH, Smeeth L, Miranda JJ. Migration surrogates and their association with obesity among within-country migrants. Obesity (Silver Spring) 2010; 18(11): 2199–2203. 20395946 10.1038/oby.2010.92 PMC3000553

[bib34] Kontis V, Mathers CD, Rehm J, Stevens GA, Shield KD, Bonita R et al. Contribution of six risk factors to achieving the 25x25 non-communicable disease mortality reduction target: a modelling study. Lancet 2014; 384(9941): 427–437. 24797573 10.1016/S0140-6736(14)60616-4

[bib35] Egan BM, Lackland DT, Jones DW. Prehypertension: an opportunity for a new public health paradigm. Cardiol Clin 2010; 28(4): 561–569. 20937441 10.1016/j.ccl.2010.07.008

[bib36] Francis ER, Kuo CC, Bernabe-Ortiz A, Nessel L, Gilman RH, Checkley W et al. Burden of chronic kidney disease in resource-limited settings from Peru: a population-based study. BMC Nephrol 2015; 16: 114. 26205002 10.1186/s12882-015-0104-7 PMC4512019

