# Supplementary material for: Rural-to-urban migration and risk of hypertension: longitudinal results of the PERU MIGRANT study
Source: J Hum Hypertens. 2016 Feb 11;31(1):22–8. doi: 10.1038/jhh.2015.124 (PMC4981561; doi:10.1038/jhh.2015.124)
Supplement: Supplementary Information [file jhh2015124x1.doc]

# ONLINE SUPPLEMENT

**E-Table 1:** Follow-up characteristics of the study sample according to study group: analysis including those without hypertension at baseline

|  |  | **Rural group** | **Migrant group** | **Urban group** | **p-value** |
| --- | --- | --- | --- | --- | --- |
|  |  | **(n = 169)** | **(n = 463)** | **(n = 130)** |
| ***Age (years)**** | Mean (SD) | 51.8 (11.4) | 51.2 (10.5) | 49.3 (9.6) | 0.11 |
| ***Systolic blood pressure (mmHg)**** | Mean (SD) | 107.4 (13.5) | 109.9 (13.2) | 110.0 (13.9) | 0.12 |
| ***Diastolic blood pressure (mmHg)**** | Mean (SD) | 67.6 (8.7) | 67.3 (8.3) | 67.2 (7.9) | 0.92 |
| ***Blood pressure status*** | Normal | 103 (70.1%) | 336 (74.0%) | 102 (82.3%) | 0.01 |
|  | Pre-hypertension | 21 (14.3%) | 82 (18.1%) | 15 (12.1%) |  |
|  | Hypertension | 23 (15.6%) | 36 (7.9%) | 7 (5.6%) |  |

Results may not add due to missing values.

* Analysis of variance test was used for comparisons, instead of Chi squared test for categorical variables

**E-Table 2:** Factors and risk of hypertension according to study group: Population attributable fractions

|  | **Rural group** | | **Migrant group** | | **Urban group** | |
| --- | --- | --- | --- | --- | --- | --- |
|  | **RR*** | **PAF** | **RR*** | **PAF** | **RR*** | **PAF** |
| **Binge drinking** |  |  |  |  |  |  |
| Yes | 1.33 | 3.3% | 0.57 | -2.1% | -- | -- |
| **Current daily smoking** |  |  |  |  |  |  |
| Yes | 4.26^†^ | 3.3% | -- | -- | -- | -- |
| **Physical activity** |  |  |  |  |  |  |
| Low levels | -- | -- | 0.87 | -4.2% | 0.90 | -3.1% |
| **High total cholesterol** |  |  |  |  |  |  |
| ≥ 200 mg/dL | 2.34 | 10.0% | 1.27 | 10.0% | 1.05 | 2.0% |
| **Obesity** |  |  |  |  |  |  |
| BMI ≥ 30 kg/m^2^ | 2.39 | 5.1% | 1.15 | 3.6% | 3.79 | 52.6% |
| **High waist circumference** |  |  |  |  |  |  |
| Yes | 2.68^†^ | 19.1% | 1.56 | 27.9% | 2.15^†^ | 45.8% |
| **Type 2 diabetes** |  |  |  |  |  |  |
| Yes | **--** | **--** | 1.70 | 3.4% | 7.10^†^ | 24.5% |
| **Pre-hypertension** |  |  |  |  |  |  |
| Yes | 2.24 | 31.3% | 2.98^†^ | 40.6% | 2.98 | 37.9% |

* Adjusted RR obtained in previous models (Table 4) was used in the calculation of population attributable risk for each risk factor.

^†^ Relative risk was significant in the multivariable model.
